# Supplementary material for: Growth Cost and Transport Efficiency Tradeoffs Define Root System Optimization Across Varying Developmental Stages and Environments in Arabidopsis
Source: bioRxiv. 2025 Jul 26:2025.07.25.666579. Preprint. [Version 1] doi: 10.1101/2025.07.25.666579 (PMC12330678; doi:10.1101/2025.07.25.666579)
Supplement: Supplement 2 — Supplemental 1: Evaluation of the trained models [file media-2.docx]

Evaluation of the Trained Models

For all models we use Mean Squared Error (MSELoss) as the loss function during training. For evaluation, we compute Mean Absolute Error (MAE), Root Mean Squared Error (RMSE) and R-squared (R^2^) score.

1. Total Root Length


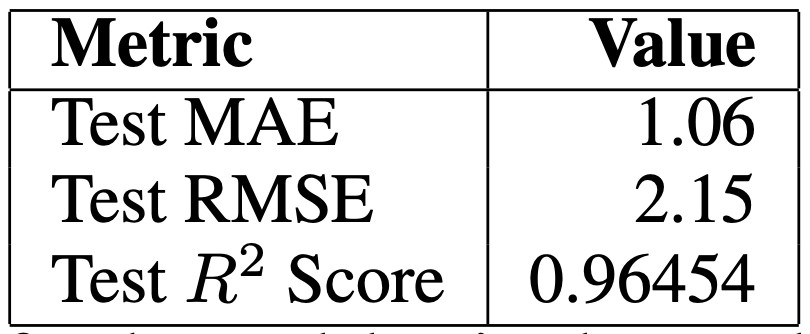


Table 1: Test metrics for the model trained to predict Total Root Length

2. Alpha


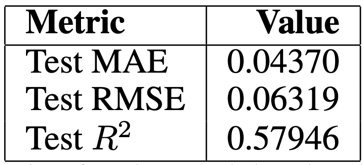


Table 2: Test metrics for the model trained to predict alpha
