# Supplementary material for: Growth Cost and Transport Efficiency Tradeoffs Define Root System Optimization Across Varying Developmental Stages and Environments in Arabidopsis
Source: bioRxiv. 2025 Jul 26:2025.07.25.666579. Preprint. [Version 1] doi: 10.1101/2025.07.25.666579 (PMC12330678; doi:10.1101/2025.07.25.666579)
Supplement: 7 [file NIHPP2025.07.25.666579V1-supplement-7.pdf]

bioRxiv preprint doi: https://doi.org/10.1101/2025.07.25.666579; this version posted July 26, 2025. The copyright holder for this preprint (which was not certified by peer review) is the author/funder, who has granted bioRxiv a license to display the preprint in perpetuity. It is made available under aCC-BY 4.0 International license.

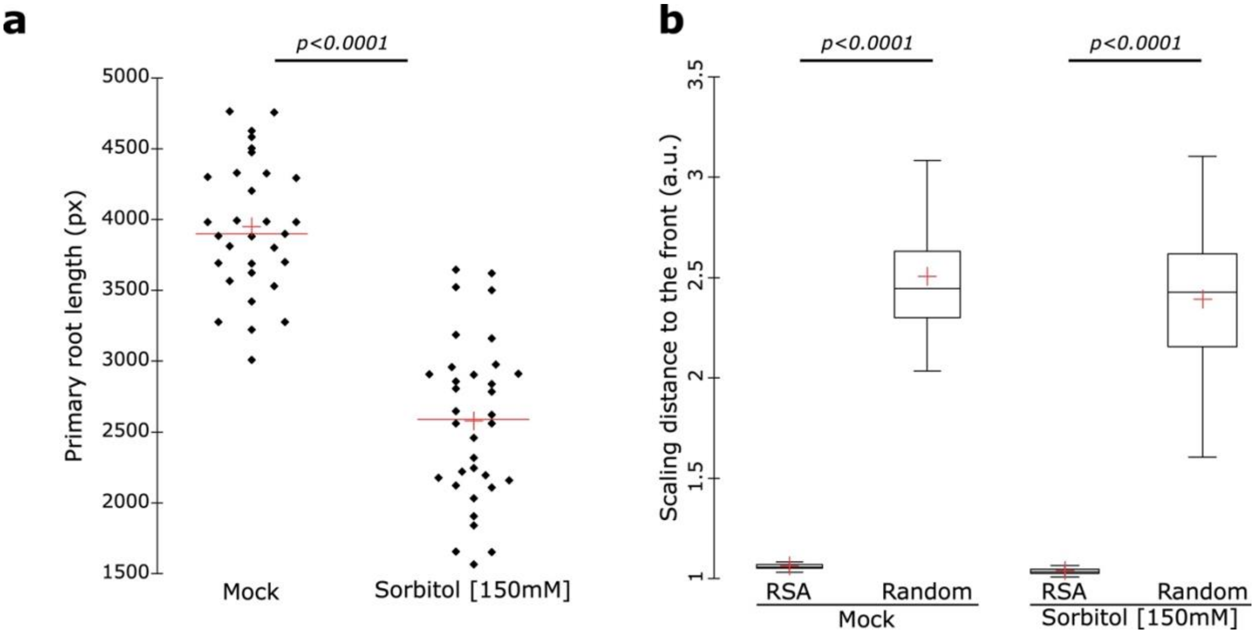

Figure S1: Osmotic stress induces a root growth decrease. a, graph depicting the primary root length of plants grown under control and sorbitol at 150mM. [Two-ways Student t-test, (p=0.05)]. **b**, graph depicting the scaling distance to the front in mock and Sorbitol conditions for RSA and random architectures. [Two-ways Student t-test (p=0.05)]. The red crosses depict the mean and the red bars the median.

bioRxiv preprint doi: https://doi.org/10.1101/2025.07.25.666579; this version posted July 26, 2025. The copyright holder for this preprint (which was not certified by peer review) is the author/funder, who has granted bioRxiv a license to display the preprint in perpetuity. It is made available under aCC-BY 4.0 International license.

a

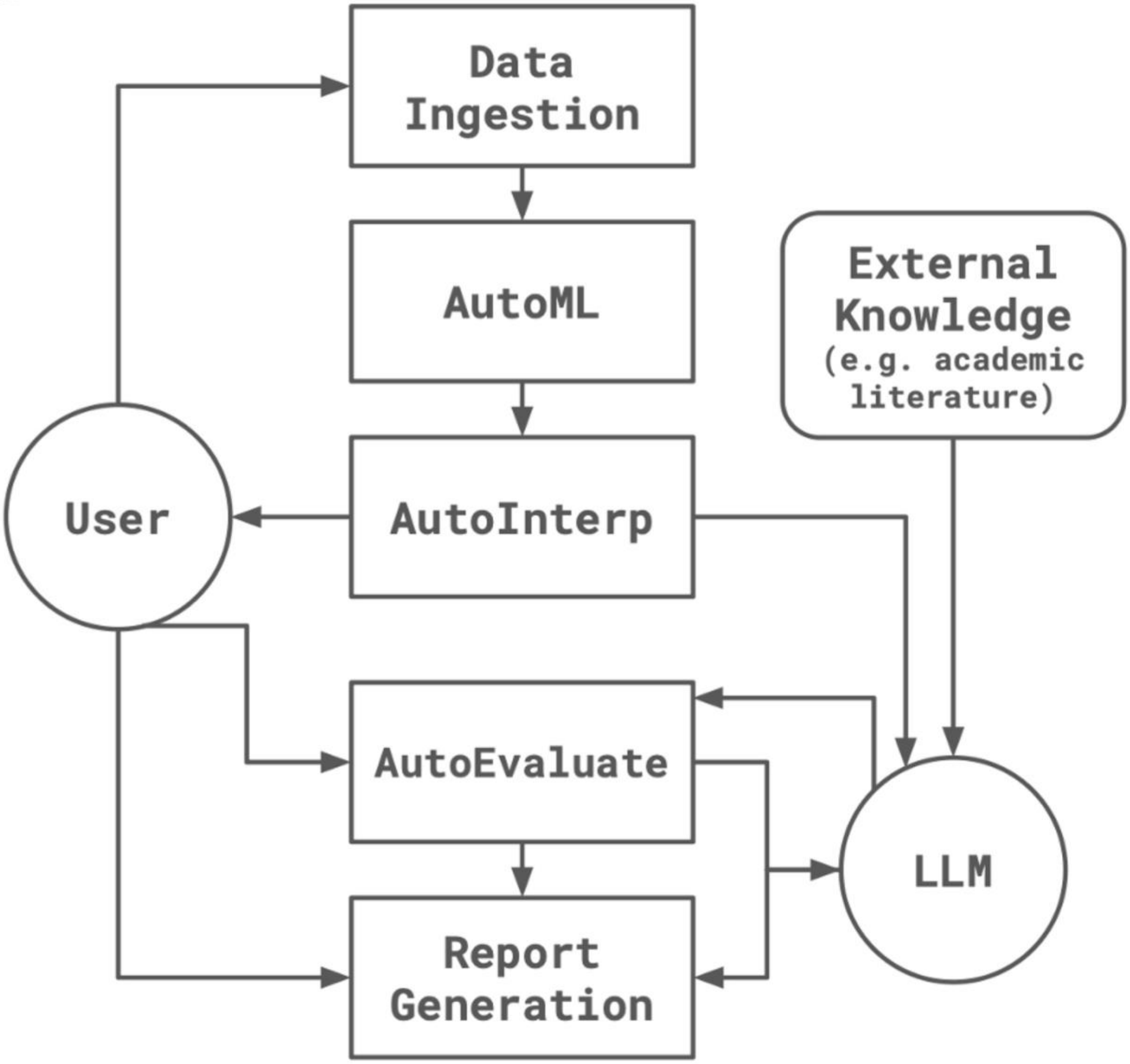

Figure S2: Schematic of the Discovery Engine. a, Schematic representation of the Discovery Engine and the different steps

bioRxiv preprint doi: https://doi.org/10.1101/2025.07.25.666579; this version posted July 26, 2025. The copyright holder for this preprint (which was not certified by peer review) is the author/funder, who has granted bioRxiv a license to display the preprint in perpetuity. It is made available under aCC-BY 4.0 International license.

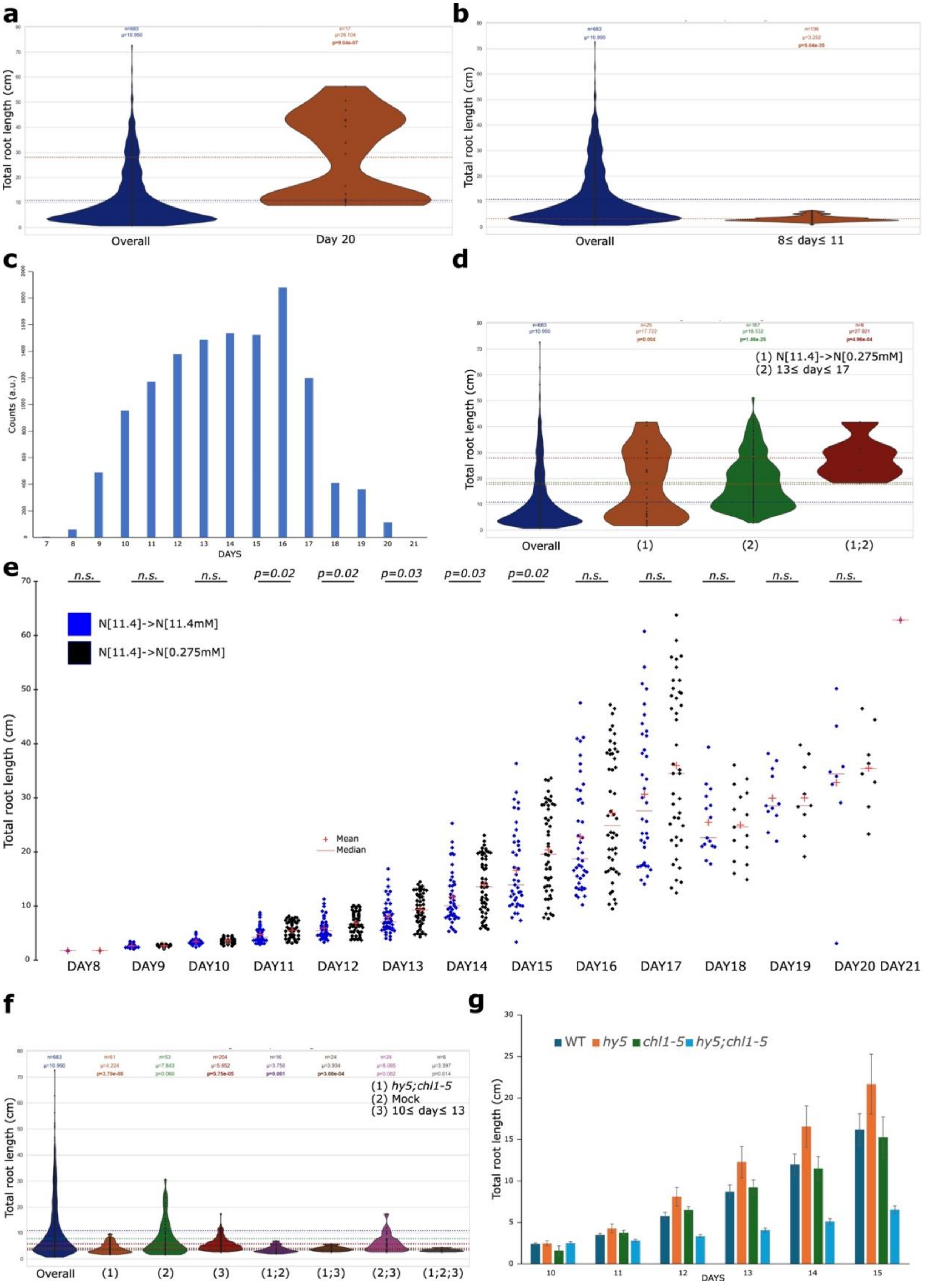

bioRxiv preprint doi: https://doi.org/10.1101/2025.07.25.666579; this version posted July 26, 2025. The copyright holder for this preprint (which was not certified by peer review) is the author/funder, who has granted bioRxiv a license to display the preprint in perpetuity. It is made available under aCC-BY 4.0 International license.

Figure S3: Analysis of the raw data for the total root length. **a**, Quantification of the total root length throughout the entire dataset (Overall) and at day 20. **b**, Quantification of the total root length throughout the entire dataset (Overall) and between day 8 to 11. **c,** Quantification of the number of counts per day in the entire dataset. **d**, Quantification of the total root length throughout the entire dataset (Overall), for plants grown for 5 days on agar plates under 11.4mM of N then transferred to agar plates containing 0.275mM of N and for plants between day 8 to 11 and their different combinations. **e,** Quantification of the total root length for plants grown for 5 days on agar plates under 11.4mM of N then transferred to agar plates containing 0.275mM or 11.4mM of N per day. **f**, Quantification of the total root length throughout the entire dataset (Overall), for the *hy5;chl5-1* mutant, for plants grown under mock conditions, for plants between day 10 to 13 and their different combinations. **g**, Quantification of the total root length per day for the indicted genotypes in mock conditions. [Two-ways Student t-test, (p=0.05)]. n, depicts the number of samples, μ, the mean and p, the p-value compared the overall group. The red crosses depict the mean and the red bars the median.

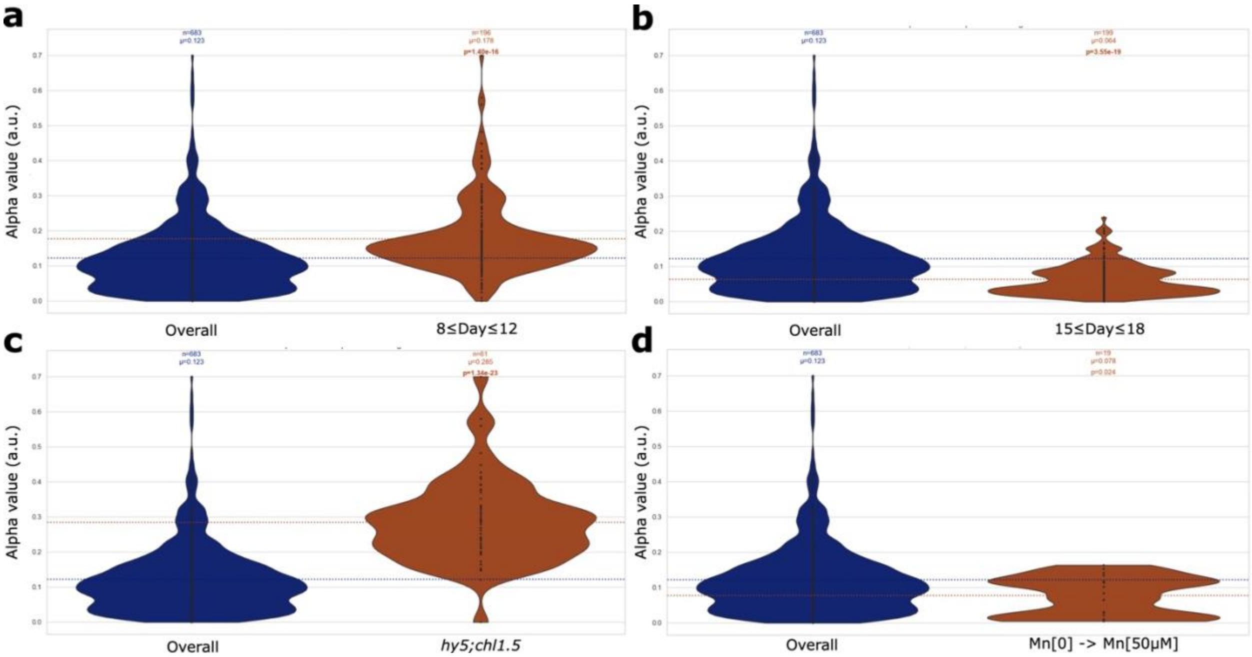

Figure S4: Analysis of the raw data for the alpha value. **a**, Quantification of the alpha value throughout the entire dataset (Overall) and between day 8 to 12. **b**, Quantification of the alpha value throughout the entire dataset (Overall) and between day 15 to 18. **c**, Quantification of the alpha value throughout the entire dataset (Overall) or for the *hy5;chl5-1* mutant. **d**, Quantification of the alpha value throughout the entire dataset (Overall), for plants grown for 5 days on agar plates under 0μM of Mn then transferred to agar plates containing 50μM of Mn. [Two-ways Student t-test, (p=0.05)]. n, depicts the number of samples, μ, the mean and p, the p-value compared the overall group.

bioRxiv preprint doi: https://doi.org/10.1101/2025.07.25.666579; this version posted July 26, 2025. The copyright holder for this preprint (which was not certified by peer review) is the author/funder, who has granted bioRxiv a license to display the preprint in perpetuity. It is made available under aCC-BY 4.0 International license.

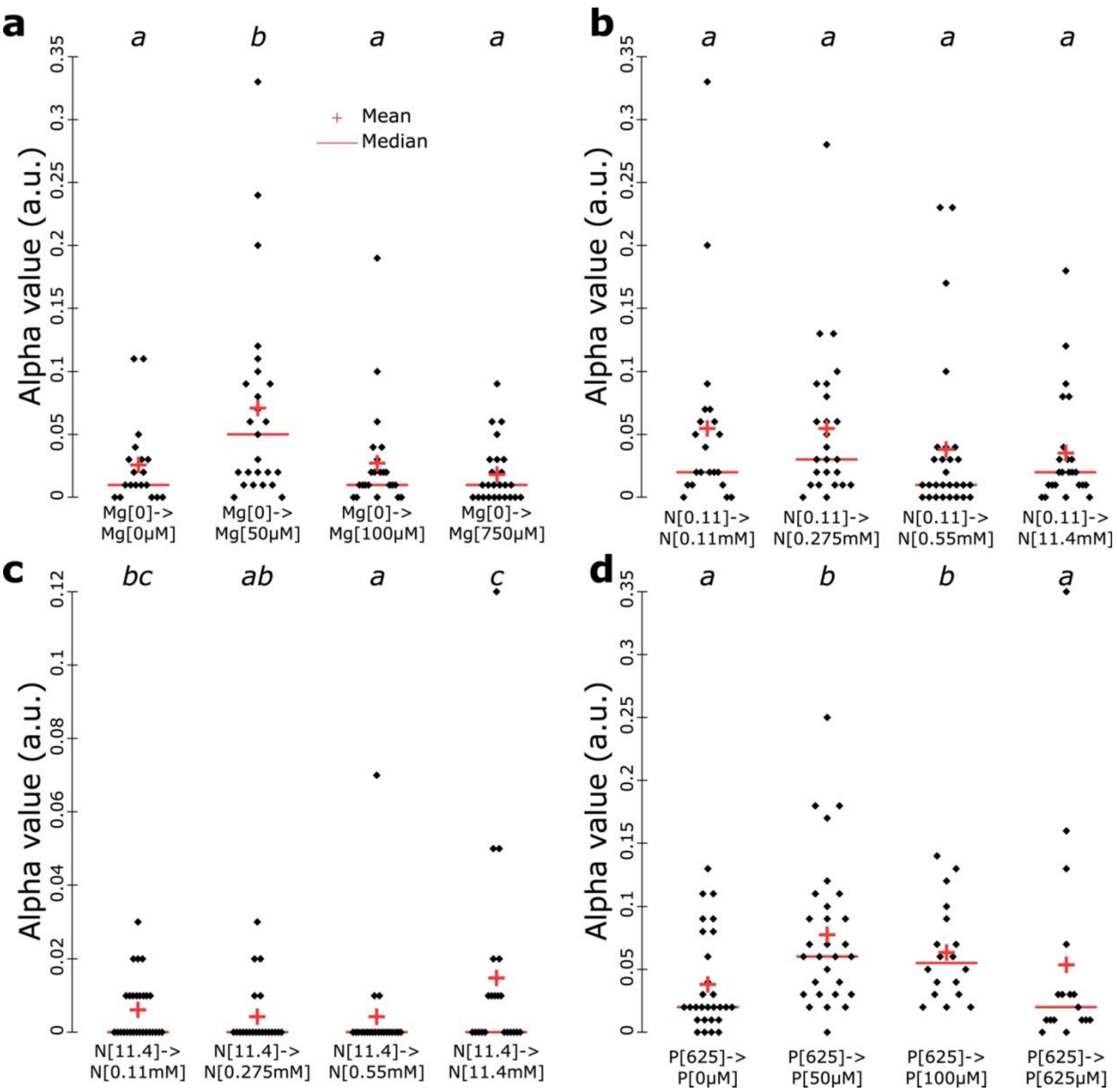

Figure S5: The Mg, P and N does not modulate the alpha value in a concentration-dependent manner. a, Quantification of the alpha value for 17-day-old plants grown for 5 days on agar plates under 0µM of Mg then transferred to agar plates containing 0, 50, 100 and 750µM of Mg. **b**, Quantification of the alpha value for 17-day-old plants grown for 5 days on agar plates under 0.11mM of N then transferred to agar plates containing 0.11, 0.275, 0.550 and 11.4mM of N. **c**, Quantification of the alpha value for 17-day-old plants grown for 5 days on agar plates under 11.4mM of N then transferred to agar plates containing 0.11, 0.275, 0.550 and 11.4mM of N. **d**, Quantification of the alpha value for 17-day-old plants grown for 5 days on agar plates under 625µM of P then transferred to agar plates containing 0, 50, 100 and 625µM of P. [two-way Kruskal-Wallis coupled with post hoc Steel-Dwass-Critchlow-Fligner procedure was performed, letters indicate statistical differences ($p < 0.05$)]. The red crosses depict the mean and the red bars the median.
